# Childhood Emotional Maltreatment and Romantic Relationships: The Role of Compassionate Goals

**DOI:** 10.3389/fpsyg.2021.723126

**Published:** 2021-11-29

**Authors:** Lining Sun, Amy Canevello, Kathrine A. Lewis, Jiqiang Li, Jennifer Crocker

**Affiliations:** ^1^Department of Psychology, National University of Singapore, Singapore, Singapore; ^2^Department of Psychology, The Ohio State University, Columbus, OH, United States; ^3^Department of Psychological Science, University of North Carolina at Charlotte, Charlotte, NC, United States; ^4^Department of Psychology, The Pennsylvania State University, University Park, PA, United States

**Keywords:** relationship quality, childhood emotional maltreatment, compassionate goals, romantic partner, interpersonal goals

## Abstract

Past research indicates that childhood emotional maltreatment (CEM) undermines the quality of adult romantic relationships by fostering negative characteristics in survivors. Two longitudinal studies investigated the hypothesis that decreased compassionate goals toward partners over time explain the association between CEM and declining relationship quality. In Study 1, CEM predicted decreased compassionate goals over time, which in turn predicted decreased relationship quality in individuals in romantic relationships. Study 2 replicated this effect in romantically involved couples and showed that partners’ high compassionate goals attenuated the decline in compassionate goals associated with reported CEM. These results point to the importance of examining how CEM may affect positive relationship processes and the protective roles of partners’ compassionate goals.

## Introduction

Childhood adversity undermines well-being in adulthood (Edwards et al., 2003). Abuse and neglect are particularly traumatic forms of childhood adversity, and can include physical, sexual, and emotional maltreatment. Childhood emotional maltreatment (CEM) is a prevalent yet under-investigated type of childhood adversity (Scher et al., 2004). CEM refers to abuse and neglect where a child’s basic emotional needs are consistently unmet (Hart and Brassard, 1987). CEM relates to a range of serious cognitive, emotional, and behavioral consequences in adulthood (Briere and Runtz, 1990; Mullen et al., 1996; Angelakis et al., 2019). For example, people with CEM report more depression (see Nelson et al., 2017, for a review), more suicidal ideation and suicide attempts (see Angelakis et al., 2019, for a review), and they are more likely to suffer from multiple mental disorders in adulthood (Taillieu et al., 2016). Parents who report having experienced CEM tend to be more hostile toward their children, increasing the likelihood of psychologically maltreating their children and creating a cycle of abuse (Bailey et al., 2012).

Although existing research has documented multiple detrimental effects of CEM on individuals, limited evidence indicates that CEM predicts impaired functioning in romantic relationships in adulthood. More attention to this issue is warranted because high quality relationships, particularly intimate relationships, predict health and well-being. For example, a meta-analysis of 148 studies (308,849 participants) found that stronger relationships are associated with decreased mortality, with effect sizes similar to quitting smoking (Holt-Lunstad et al., 2010). Likewise, high-quality close relationships are among the strongest predictors of happiness and psychological well-being (Myers and Diener, 1995). For many people, romantic and marital relationships are their closest and most enduring relationships (Chonody and Gabb, 2019). Thus, understanding the effects of CEM on the quality of romantic relationships may contribute to improvements in their well-being.

### Relationship Quality Over Time

Relatively little research has examined how relationship quality changes over time in romantic relationships. Theoretical perspectives make differing predictions regarding the course of relationship quality over time (see Sprecher, 1999, for a discussion). Some theories suggest that people may habituate to the positive aspects of romantic relationships, leading them to become less rewarding over time (e.g., Huesmann, 1980; Aron and Aron, 1986). Other perspectives propose that relationship quality increases as relationships advance from less committed (e.g., dating relationships) to more committed (e.g., marriage). Still other perspectives predict an initial increase in relationship quality in the early stages of a romantic relationship due to the rewarding and self-expanding qualities of a new relationship, which may then level off or decline in some relationships, leading to dissolution. In sum, theoretical perspectives allow for a wide range of trajectories in the quality of romantic relationships over time.

Studies of the trajectories of romantic relationships often observe relatively high levels of relationship quality that declines over time (VanLaningham et al., 2001; DiLillo et al., 2009). One study particularly relevant to the present investigation examined changes in relationship quality among 101 heterosexual dating couples at five times over 4 years (Sprecher, 1999). Over the 4 years of the study, 59% of the couples had ended their relationships. Participants whose relationships were still intact at each wave of the study perceived that their positive feelings (love, commitment, and satisfaction) had increased over time, although contemporaneous reports showed declines in positive feelings over the first 6 months of the study and very little change in these positive feelings thereafter. The one exception for couples who stayed together for the full 4 years of the study (79% of whom were married at that point) was that women’s commitment and satisfaction increased from wave 4 to wave 5 of the study. In contrast to intact couples, those whose relationships broke up over the course of the study perceived that their positive feelings had declined over time.

Thus, dating relationships that eventually break up are, unsurprisingly, characterized by declining positive feelings over time. Perhaps more surprising, declines in positivity may be more important than negativity. In interpersonal relationships, negative and positive processes are functionally distinct (Reis and Gable, 2003). For example, positivity declines while levels of negativity remain rather stable in relationship trajectories (Huston et al., 2001). Decreased positivity is more strongly associated with relationship dissolution than the emergence of negativity (Huston et al., 2001). As Huston and Vangelisti (1991, p. 721) put it, “People who might have been on their best behavior while courting may settle into behavior that reflects their more stable, underlying dispositions and attitudes,” leading to a decline in relationship quality over time.

### Childhood Emotional Maltreatment and Romantic Relationships

Research shows that adults who retrospectively report more childhood maltreatment have more dysfunctional relationships (DiLillo et al., 2009). People who report CEM (referred to as “CEM survivors” or “survivors” hereafter) have relatively poor quality romantic relationships. CEM survivors tend to feel less safe and more reluctant to enter into adult relationships (Kapeleris and Paivio, 2011). They report lower trust (DiLillo et al., 2009), more conflict (Briere and Rickards, 2007), higher relationship dissatisfaction (Perry et al., 2007; Maneta et al., 2015; Liu et al., 2019), and a greater likelihood of relationship dissolution (Mullen et al., 1996). In a longitudinal study of newlywed couples, DiLillo et al. (2009) found that reports of childhood maltreatment predicted lower relationship satisfaction in both husbands and wives. Furthermore, the negative effects of maltreatment on marital functioning became stronger over time, particularly for husbands.

Most investigations aiming to understand why CEM is associated with poor and declining relationship quality have focused on negative characteristics of survivors, such as insecure attachment (Riggs et al., 2011; Lassri et al., 2016), hostility (Perry et al., 2007), and depressive symptoms (Perry et al., 2007; Wright et al., 2009). Although this research has been informative, it neglects the possibility that CEM can undermine positive processes that foster high relationship quality and sustain it over time. As noted, positive processes are functionally distinct from negative processes, and declines in positive processes are linked with declining relationship quality and relationship dissolution. If CEM negatively affects positive relationship processes this may help explain CEM survivors’ poor relationship quality and potentially their declining relationship quality over time.

Indeed, due to their history of abuse and neglect, CEM survivors’ relationships may be characterized by less positivity initially, and steeper declines in positive relationship processes over time, relative to people who do not report experiencing CEM. In the terminology of Huston and Vangelisti (1991), the underlying dispositions of CEM survivors may lead to patterns that are characterized by particularly low positivity.

### Childhood Emotional Maltreatment and Compassionate Goals

Among the positive mechanisms that CEM may impair are prosocial motivations such as compassionate goals. Compassionate goals are intentions to support the well-being of others and not harm them (Crocker and Canevello, 2008). Compassionate goals foster a supportive interpersonal environment. When people have compassionate goals, they tend to feel more peaceful, clear, connected, and secure in their relationships (Crocker and Canevello, 2008). They hold a non-zero-sum and cooperative mindset toward relationships (Canevello and Crocker, 2017; Crocker et al., 2017), and become more supportive and responsive toward relationship partners over time (e.g., Crocker and Canevello, 2008; Canevello and Crocker, 2010). In turn, their partners tend to notice and reciprocate these constructive behaviors (Crocker and Canevello, 2008; Canevello and Crocker, 2010). The resulting upward spirals of responsiveness predict improved relationship quality and well-being for both people (see Crocker and Canevello, 2012, 2016, for reviews). Thus, compassionate goals contribute to high-quality romantic relationships, especially in the face of challenges and difficulties (Crocker and Canevello, 2016; Crocker et al., 2017).

Compassionate goals may explain why CEM survivors struggle to build and sustain high quality romantic relationships. We propose that CEM impairs survivors’ ability to have and sustain compassionate goals toward their romantic partners, even those whom they care for and believe care for them. That is, CEM survivors may have compassionate goals early on in their romantic relationships (albeit less than those who did not experience CEM). However, due to their history of emotional maltreatment, particularly experiences with caregivers who were unresponsive to their needs (Hart and Brassard, 1987), they may be less responsive and perceive their partners as less responsive (i.e., manifesting lower levels in understanding, caring, and validation) (Kochanska and Aksan, 2004; Lumley and Harkness, 2007; Canevello and Crocker, 2010). Experiencing relationships characterized with low responsiveness could decrease CEM survivors’ intentions to be constructive and supportive to partners (i.e., compassionate goals) over time. This decline in compassionate goals may account for a concurrent decline in relationship quality. Thus, whereas people with high compassionate goals can create upward spirals of responsiveness and relationship quality in their relationships, CEM survivors may create downward spirals due to their lower levels of compassionate goals, which decline more over time, undermining relationship quality.

Thus, we hypothesized that CEM survivors’ compassionate goals tend to decline more over time than is the case for people who did not experience CEM. Based on previous research (Canevello and Crocker, 2010), we further hypothesized that decreased compassionate goals predict simultaneous deterioration of relationship quality.

Because positive and negative processes in close relationships can be independent of one another, we hypothesized that these changes over time in the relationships of CEM survivors are independent of other mechanisms known to be associated with poor relationship functioning in those with a history of CEM (i.e., attachment anxiety and avoidance, hostility, and depression).

### The Role of Partners

Romantic partners may have an important role in the process by which CEM predicts declines in relationship quality via decreased compassionate goals. Partners’ negative characteristics (e.g., depression and aggression) do not appear to moderate the effects of childhood abuse on adult romantic relationship quality (Nguyen et al., 2017). However, other research suggests that partners’ positive characteristics and behavior may play a positive role in childhood maltreatment survivors’ lives. For example, partners’ positive social support facilitates male physical abuse survivors’ posttraumatic recovery (Evans et al., 2014). As noted, maltreatment survivors tend to assume that others will not understand, care for, or validate them (Lumley and Harkness, 2007). When partners have compassionate goals toward CEM survivors, survivors’ negative expectations about their partners may be challenged, interrupting the decline in survivors’ compassionate goals and the downward spiral in their relationships. Accordingly, we also hypothesize that partners’ higher compassionate goals can attenuate the hypothesized decline in CEM survivors’ compassionate goals over time. Thus, partners with higher compassionate goals may interrupt the downward spiral in CEM survivors’ relationships.

### Summary of Hypotheses

In sum, we had two main hypotheses. Our first hypothesis had three parts: (1) those who report more CEM have difficulty sustaining compassionate goals toward their romantic partners, and therefore show declines in compassionate goals over time; (2) that this decline in compassionate goals toward partners mediates (i.e., accounts for) concurrent declines in their relationship quality; and (3) that these declines in compassionate goals and relationship quality are independent of several possible alternative explanations for the association, including survivors’ attachment anxiety and avoidance, hostility, and depression. Our second main hypothesis was that the association between CEM and declines in compassionate goals is moderated by partners’ compassionate goals, such that when partners have more compassionate goals, the negative association between CEM and change in compassionate goals is attenuated.

### The Present Studies

We conducted two longitudinal studies to test these hypotheses. Study 1 tested the first hypothesis in a sample of individuals in romantic relationships who completed measures at two times, approximately 2 months apart. Study 2 tested whether the predicted association between CEM and declining compassionate goals depends on partners’ compassionate goals in a sample of romantically involved couples. We assessed gender, socially desirable responding, attachment anxiety and avoidance, hostility, and depression to rule out alternative explanations of our findings suggested by previous research (Perry et al., 2007; Wright et al., 2009; Riggs et al., 2011; Lassri et al., 2016).

We also explored whether relationship length moderates the association between CEM and change in compassionate goals. We did not anticipate moderating effects, because compassionate goals can be found at any stage of relationships and in many different types of relationships, including relationships with friends, new college roommates, and romantic partners^1^, ^2^. Nonetheless, to address the possibility that associations observed in these studies depend on relationship length, we tested moderation effects.

Finally, because many romantic relationships break up over time and this is particularly true for people who report CEM (Perry et al., 2007; Maneta et al., 2015; Liu et al., 2019), we explored whether CEM predicts breakups in Study 1, and whether declines in compassionate goals account for this association.

## Study 1

### Method

#### Participants

One hundred and sixty-five individuals (37 males, 125 females, and 3 who did not provide gender information) currently in romantic relationships were recruited for a study of relationships and health. They ranged in age from 18 to 34 years (*M* = 19.45, *SD* = 2.34). Relationship length ranged from 18 days to 11.36 years (*M* = 1.53 years, *SD* = 1.57). Participants received credit toward their introduction to psychology course (44.8%), pay (27.9%), or a combination of credit and pay (27.3%). Paid participants received $5 and $20, respectively, for completing an initial online survey and each of two follow-up surveys. Of the sample, 81.8% were European American/White, 12.1% were Asian, 7.3% were African American/Black, 1.2% were Native American/Alaska Native; 6.7% self-identified as Hispanic/Latino of any race. One hundred and forty-three participants (87%) completed a 2-month follow-up survey. This sample size provides more than 80% power to detect an effect size of *f*^2^ = 0.06 (Perry et al., 2007) at α = 0.05 according to G^∗^Power 3.1 (Faul et al., 2007). The study was approved by an Institutional Review Board at The Ohio State University.

#### Procedure

Participants completed measures at three times: in an online pretest, in an initial laboratory session (T1; approximately 2 days after the pretest), and in a second laboratory session (T2; approximately 2 months after T1). In the online pretest, participants completed measures of CEM, socially desirable responding, hostility, and depression. At T1, participants came to the laboratory to complete measures of compassionate goals, relationship quality, and attachment anxiety and avoidance. At T2, participants returned to the laboratory to complete a follow-up measure of compassionate goals (*N* = 143). Those still with their romantic partners (*N* = 132) completed a second measure of relationship quality (see Canevello and Crocker, 2017; Crocker et al., 2017, for other findings from this study).

#### Primary Measures

##### Childhood Emotional Maltreatment

The Childhood Trauma Questionnaire (CTQ; Bernstein and Fink, 1998) assesses self-reports of maltreatment experienced during childhood. Participants rated ten statements from 1 (*never true*) to 5 (*very often true*) regarding their families prior to age 13, half on emotional abuse (e.g., “People in my family said hurtful or insulting things to me”; *M* = 7.61, *SD* = 3.82, range = 5–24) and the rest on emotional neglect [e.g., “People in my family looked out for each other” (reverse scored); *M* = 7.92, *SD* = 3.26, range = 5–19]. We averaged emotional abuse and neglect scores (*r* = 0.62, *p* < 0.001; Lassri et al., 2016). Because CEM was skewed (skewness = 1.70, *SE* = 0.20, kurtosis = 2.79, *SE* = 0.40), we applied a square root transformation and standardized CEM scores (Lassri et al., 2016).

##### Compassionate Goals

As in previous research (Crocker et al., 2017), compassionate goals toward romantic partners were assessed with 8 items (e.g., “be supportive of my partner;” “have compassion for my partner’s mistakes and weaknesses”). Items began with the phrase, “Over the past 2 weeks, in my romantic relationship, I wanted/tried to…” and were rated on a 5-point scale (1 = *not at all*; 5 = *extremely*).

##### Relationship Quality

At T1 and T2, participants rated their satisfaction, commitment, and closeness in the relationship. Satisfaction was measured with six items (e.g., “We had a good relationship”) from the relationship satisfaction scale (Norton, 1983; see Crocker et al., 2017; 1 = *strongly disagree*, 5 = *strongly agree*). Commitment was measured with four items (e.g., “Do you feel committed to maintaining your relationship with your partner?”; Rusbult et al., 1998; 1 = *not at all*, 8 = *completely*). Closeness was measured with two items: “How close did you feel to your partner?” and “Relative to what you know about *other people’s* romantic relationships, how would you characterize your relationship with your partner?” (1 = *not at all/not as close as others*; 5 = *extremely/much closer than others*).

Exploratory factor analyses indicated that the three scale scores loaded on one factor (all T1 loadings > 0.63; all T2 loadings > 0.60), so we combined them into an index of relationship quality at T1 and T2 by standardizing and averaging the three scale scores for each time-point (Canevello and Crocker, 2010).

##### Covariates

*Attachment Anxiety and Avoidance* over the past 2 weeks were assessed at T1 with an 18-item version of the Experiences in Close Relationships Questionnaire (ECR; Brennan et al., 1998; Canevello et al., 2013). Items were rated on a 5-point scale (1 = *strongly disagree*; 5 = *strongly agree*).

*Hostility* was measured in the online pretest with 8 items from the Buss and Perry Aggression Questionnaire (BPAQ; Buss and Perry, 1992). Items were rated on a 5-point scale (1 = *not at all characteristic*; 5 = *very characteristic*).

*Depression* over the past 2 weeks was assessed using the 20-item CES-D Scale (Radloff, 1977). Items were rated on a 5-point scale [1 = *Rarely* (*Less than 1 day*), 2 = *Occasionally* (*1–2 days*), 3 = *Often* (*3–4 days*), 4 = *Almost always* (*5–7 days*); 5 = *Always* (*7–14 days*)].

*Socially Desirable Responding* was assessed with the scale developed by Crowne and Marlowe (1960). Participants answered *Yes/No* to 33 items.

### Results

#### Overview of Analyses

After conducting descriptive analyses, we standardized all variables to obtain interpretable and comparable effect sizes (Schielzeth, 2010)^3^. We created residual change scores for compassionate goals and relationship quality by regressing the T2 variable on the T1 variable and saving the residuals as the indicator of residual change.

We examined the association between CEM and change in compassionate goals from T1 to T2 and whether this association could be explained by social desirability, initial relationship quality, attachment anxiety and avoidance, hostility, and depression. Next, we tested a model in which CEM predicted change in compassionate goals from T1 to T2, which in turn predicted change in relationship quality from T1 to T2. We then repeated this analysis with attachment anxiety and avoidance, hostility, and depression entered as simultaneous mediators to test whether the indirect effect through compassionate goals remained significant when we controlled for these negative mechanisms. We also examined whether this mediation model depended on (i.e., was moderated by) relationship length. Finally, we conducted exploratory analyses to examine whether decreased compassionate goals mediated the association between CEM and relationship dissolution.

Table 1 presents the means, standard deviations, coefficient alphas, and correlations among variables. As predicted, CEM correlated negatively with compassionate goals and relationship quality at both time points. Consistent with past research (Canevello and Crocker, 2010, Study 2), compassionate goals correlated positively with relationship quality at T1 and T2. Because gender was not related to any other variables, it was not included in the subsequent analyses.

**TABLE 1:** Means, standard deviations, coefficient alphas, and correlations among Study 1 variables.

		***M***	***SD***	**α**	**1**	**2**	**3**	**4**	**5**	**6**	**7**	**8**	**9**	**10**	**11**
(1)	CEM^*a*^	−	−	0.76											
(2)	Compassionate goals (T1)	4.18	0.53	0.80	−0.21^*^										
(3)	Compassionate goals (T2)	4.03	0.70	0.86	−0.32^***^	0.53^***^									
(4)	Relationship quality (T1^a^)	−	−	0.76	−0.34^***^	0.37^***^	0.39^***^								
(5)	Relationship quality (T2^a^,^b^)	−	−	0.89	−0.15	0.34^***^	0.52^***^	0.54^***^							
(6)	Attachment Anxiety (T1)	2.16	0.87	0.87	0.30^***^	−0.17^*^	−0.16	−0.52^***^	−0.19^*^						
(7)	Attachment Avoidance (T1)	1.56	0.56	0.87	0.16	−0.40^***^	−0.38^***^	−0.56^***^	−0.30^***^	0.42^***^					
(8)	Hostility (online pretest)	2.24	0.80	0.84	0.17^*^	−0.30^***^	−0.21^*^	−0.18^*^	−0.16	0.32^***^	0.26^**^				
(9)	Depression (online pretest)	1.85	0.53	0.86	0.35^***^	−0.23^**^	−0.24^**^	−0.35^***^	−0.10	0.46^***^	0.31^***^	0.47^***^			
(10)	Social desirability	16.18	4.99	0.74	−0.28^***^	0.35^***^	0.23^**^	0.22^**^	0.13	−0.17^*^	−0.18^*^	−0.46^***^	−0.21^*^		
(11)	Gender	−	−	−	0.05	−0.09	−0.05	−0.04	−0.07	0.11	0.03	0.16	0.14	0.05	
(12)	Relationship length	1.48	1.35	−	−0.08	−0.08	−0.11	0.19^*^	0.07	−0.18^*^	−0.10	−0.08	0.13	0.03	−0.004

*^a^CEM, childhood emotional maltreatment. CEM is the standardized composite of emotional abuse (M = 7.61, SD = 3.82, α = 0.86) and emotional neglect scores (M = 7.92, SD = 3.26, α = 0.86). Relationship quality at T1 and T2 are composites of standardized scores on relationship satisfaction (M_T1_ = 4.59, SD_T1_ = 0.58, α_T1_ = 0.94; M_T2_ = 4.45, SD_T2_ = 0.85, α_T2_ = 0.97), commitment (M_T1_ = 7.33, SD_T1_ = 0.97, α_T1_ = 0.78; M_T2_ = 7.23, SD_T2_ = 1.44, α_T2_ = 0.87), and closeness (M_T1_ = 4.35, SD_T1_ = 0.58, r_T1_ = 0.48; M_T2_ = 4.20, SD_T2_ = 0.82, r_T2_ = 0.68) averaged at each time. Therefore, the Ms and SDs of CEM, relationship quality at T1 and T2 were omitted.*

*Male is coded as 0 while female as 1.*

*^b^N = 143 except for relationship quality at Time 2, N = 132.*

*p < 0.05, **p < 0.01, ***p < 0.001.

#### Childhood Emotional Maltreatment as a Predictor of Change in Compassionate Goals Over Time

When we regressed residual change in compassionate goals on CEM, CEM predicted decreased compassionate goals from T1 to T2, β = −0.21, *t*(141) = −3.04, 95% CI [−0.35, −0.07], *p* = 0.003. Then we examined whether this association was due to other variables by controlling for relationship quality, attachment anxiety, attachment avoidance, hostility, depression, and social desirability at T1. The effect was significant when we included those variables together in the same analysis (β = −0.18, 95% CI [−0.34, −0.03], *p* = 0.021) as well as in separate analyses (see Supplementary Table 1.1), indicating that attachment anxiety and avoidance, hostility, depression, initial level of relationship quality, and social desirability did not account for the association between CEM and decreased compassionate goals.

#### Childhood Emotional Maltreatment as a Predictor of Change in Relationship Quality Through Change in Compassionate Goals

We tested whether residual change in compassionate goals mediated the association between T1 CEM and residual change in relationship quality on data from the 132 participants who were still with their partners at T2 using Model 4 in PROCESS (Hayes, 2013) with 10,000 bias-corrected bootstrapped samples. As hypothesized, the indirect effect (indirect effect = −0.05, 95% CI [−0.15, −0.003]), suggested that decreased compassionate goals mediated the association between T1 CEM and decreased relationship quality. The effect for the path from CEM to decreased compassionate goals was β = −0.13, *t*(130) = −1.85, 95% CI [−0.26, 0.009], *p* = 0.067; the effect for the path from decreased compassionate goals to decreased relationship quality over time was β = 0.37, *t*(129) = 4.57, 95% CI [0.21, 0.52], *p* < 0.001.

Because change in compassionate goals and change in relationship quality were assessed over the same time interval, alternative orderings of the variables in the path model are also possible. To address this, we tested an alternative model in which CEM predicted deteriorating relationship quality over time, which in turn predicted decreased compassionate goals toward partners. The alternative model was not supported; the indirect effect of CEM on change in compassionate goals was β = −0.004, 95% CI [−0.06, 0.05]. The effect of CEM on change in relationship quality was β = −0.01, *t*(130) = −0.16, 95% CI [−0.14, 0.12], *p* > 0.25.

##### The Role of Relationship Length

We explored whether relationship length moderates the link between CEM and change in compassionate goals using Model 7 in PROCESS with 10,000 bias-corrected bootstrapped samples. Relationship length did not moderate this association (interaction effect = −0.03, 95% CI [−0.18, 0.13]). The 95% confidence interval for the index of conditional mediation included 0 (index = −0.01, 95% CI [−0.07, 0.08]), suggesting that the indirect effect did not differ for longer and shorter relationships.

##### Alternative Explanations

We repeated the mediation analysis, adding attachment anxiety, attachment avoidance, hostility, and depression as simultaneous mediators with compassionate goals in a single analysis using Model 4 in PROCESS with 10,000 bias-corrected bootstrapped samples. The indirect effect through decreased compassionate goals remained (indirect effect = −0.05, 95% CI [−0.16, −0.004]). Confidence intervals for all other indirect effects included 0.

#### Childhood Emotional Maltreatment Predicting Relationship Dissolution at T2 Through Change in Compassionate Goals

The association between CEM and declining compassionate goals was based on all participants, including 11 participants who broke up with their partners between T1 and T2. When we excluded those 11 participants, the association dropped from β = −0.21 to β = −0.13, suggesting that those whose compassionate goals declined the most were also most likely to break up.

To test this possibility, we conducted an exploratory analysis examining whether change in compassionate goals explained the association between CEM and relationship dissolution at T2. Using Model 4 in PROCESS Version 2 (which accommodates dichotomous outcomes, Hayes, 2013) with 10,000 bias-corrected bootstrapped samples, the mediation model explained 35% of the variance in relationship dissolution (Nagelkerke *R*^2^). CEM predicted decreased compassionate goals (β = −0.21, *t*(141) = −3.04, 95% CI [−0.35, −0.07], *p* = 0.003). Decreased compassionate goals, in turn, predicted the increased likelihood of relationship dissolution (β = −1.30, Z = −3.62, 95% CI [−2.00, −0.60], *p* < 0.001; indirect effect = 0.27, 95% CI [0.02, 0.84]).

### Discussion

The results of Study 1 supported our hypothesis that CEM predicts decreased compassionate goals over 2 months, which in turn predicts simultaneous decreased relationship quality. Attachment anxiety and avoidance, hostility, depression, initial level of relationship quality, and social desirability did not account for the association between CEM and decreased compassionate goals. Attachment anxiety and avoidance, hostility, and depression did not account for the indirect effect of CEM on decreased relationship quality via decreased compassionate goals. Study 1 also ruled out the alternative possibility that CEM survivors have decreased relationship quality over time, which in turn predicts decreased compassionate goals.

These results are consistent with the hypothesis that CEM undermines prosocial intentions toward partners over time, which undermines relationship quality. Furthermore, exploratory analyses corroborated previous findings that CEM predicts dissolution of relationships (Mullen et al., 1996) and supported the idea that declining compassionate goals may explain this association.

## Study 2

Study 2 attempted to replicate these findings in a sample of romantic dyads. It also tested our hypothesis that partners’ compassionate goals buffer the declines in CEM survivors’ compassionate goals.

### Method

#### Participants

Eighty-three heterosexual dating couples volunteered for a study of relationships and health for credit toward their introductory psychology requirement or for pay ($40). Of these, 58 couples (116 participants) completed a 2-month follow-up survey and are included in analyses for Study 2. Two of these couples broke up during this time. Relationship length varied from 19 days to 5.3 years (*M* = 1.3 years, *SD* = 1.3). The sample ranged in age from 18 to 27 years (*M*_*Male*_ = 19.97, *SD*_*Male*_ = 1.95; *M*_*Female*_ = 19.21, *SD*_*Female*_ = 1.28). In this sample, 91.4% were European American/White, 6.9% were Asian, 2.6% were African American/Black, 1.7% were American-Indian/Alaska Native, 1.7% were Hawaiian/Pacific Islander, and 4.3% reported their race as “Other”; 6.9% self-identified as Hispanic or Latino of any race. According to Lane and Hennes (2018), this sample size exceeds that needed for power = 0.70 to detect effect sizes of β = 0.24 (Perry et al., 2007) at α = 0.05. This sample provided power of 0.80 to detect main effects of β = 0.36 at α = 0.05. The study was approved by an Institutional Review Board at The Ohio State University.

#### Procedure

The procedure for Study 2 was similar to that of Study 1 except that participants came to the laboratory together as couples at T1. Pretest measures were completed online 2 days prior to the laboratory session. Participants completed the measures separately in the T1 lab session. Participants returned to the lab after 2 months to complete the T2 follow-up survey (see Canevello and Crocker, 2017; Crocker et al., 2017, for other findings from this study).

#### Measures

Study 2 measures included childhood emotional maltreatment, compassionate goals, and covariates including attachment anxiety and avoidance, hostility, depression, and social desirability. The measures were identical to those used in Study 1^4^.

##### Childhood Emotional Maltreatment

As in Study 1, emotional abuse and neglect were highly correlated (*r* = 0.70, *p* < 0.001). Emotional abuse scores ranged from 5 to 24 (*M* = 7.24, *SD* = 3.47); emotional neglect ranged from 5 to 19 (*M* = 7.69, *SD* = 3.36). The composite CEM score was square root transformed and standardized due to skewness (skewness = 2.35, *SE* = 0.23, kurtosis = 6.30, *SE* = 0.45).

### Results

#### Overview of Analyses

Study 2 analyses addressed two main questions: (a) Does CEM predict decreased compassionate goals over 2 months, which in turn predicts decreased relationship quality as in Study 1? and (b) Do partners’ higher compassionate goals attenuate the association between CEM and decreased compassionate goals?

Specifically, Study 2 again examined the association between CEM and change in compassionate goals from T1 to T2 in a dyadic context. We also examined whether this association could be explained by social desirability, initial relationship quality, attachment anxiety and avoidance, hostility, and depression. Next, we tested a model in which CEM predicted change in compassionate goals from T1 to T2, which in turn predicted change in relationship quality from T1 to T2. We then repeated this analysis with attachment anxiety and avoidance, hostility, and depression entered as simultaneous mediators to test whether the indirect effect through compassionate goals remained significant when we controlled for these negative mechanisms. We also examined whether this mediation model depended on relationship length. Finally, we examined whether partners’ compassionate goals moderated the association between CEM and change in compassionate goals.

In these data, individuals were nested within couples. We accounted for the non-independence of individuals within dyads using the MIXED command in SPSS and treating dyad members as distinguishable by specifying heterogenous compound symmetry covariance structure (Campbell and Kashy, 2002; Kenny et al., 2006). Coefficients were derived from fixed-effects models with restricted maximum likelihood estimation and included random intercepts. All variables were measured at the individual level. All variables were standardized to provide interpretable effect sizes^5^. As in Study 1, we created residualized variables for change in compassionate goals and change in relationship quality.

Table 2 presents the means, standard deviations, coefficient alphas, and correlations for primary variables in Study 2. Because gender was not correlated with any other variables, we did not include it in subsequent analyses. Partners’ T1 compassionate goals were uncorrelated with actor variables except attachment anxiety (*r* = −0.19, *p* = 0.044).

**TABLE 2:** Means, standard deviations, coefficient alphas, and correlations among Study 2 variables.

		***M***	***SD***	**α**	**1**	**2**	**3**	**4**	**5**	**6**	**7**	**8**	**9**	**10**	**11**
(1)	CEM^*a*^	−	−	0.82											
(2)	Compassionate goals (T1)	4.24	0.48	0.82	−0.03										
(3)	Compassionate goals (T2)	4.15	0.59	0.85	−0.17	0.56^***^									
(4)	Relationship quality (T1^a^)	−	−	0.73	−0.04	0.41^***^	0.39^***^								
(5)	Relationship quality (T2^a^,^b^)	−	−	0.84	−0.02	0.37^***^	0.54^***^	0.65^***^							
(6)	Attachment anxiety (T1)	2.09	0.91	0.91	0.08	−0.16	−0.26^**^	−0.39^***^	−0.28^**^						
(7)	Attachment avoidance (T1)	1.63	0.58	0.85	0.05	−0.50^***^	−0.46^***^	−0.52^***^	−0.51^***^	0.32^***^					
(8)	Hostility (online pretest)	2.15	0.77	0.82	0.22^*^	−0.24^**^	−0.11	−0.27^**^	−0.19^*^	0.28^**^	0.22^*^				
(9)	Depression (online pretest)	1.79	0.57	0.91	0.23^*^	−0.18	−0.13	−0.22^*^	−0.29^**^	0.34^***^	0.14	0.54^***^			
(10)	Social desirability	16.64	4.85	0.75	−0.07	0.27^**^	0.10	0.27^**^	0.21^*^	−0.21^*^	−0.19^*^	−0.50^***^	−0.30^***^		
(11)	Gender	−	−	−	0.15	0.10	0.13	0.10	0.14	0.09	−0.07	−0.07	0.05	−0.06	
(12)	Relationship length	1.32	1.28	−	0.11	0.11	0.13	0.23^*^	0.24^*^	−0.02	−0.18^*^	0.15	0.17	0.10	−0.01

*^a^CEM, childhood emotional maltreatment. CEM is the standardized composite of emotional abuse (M = 7.24, SD = 3.47, α = 0.86) and emotional neglect scores (M = 7.69, SD = 3.36, α = 0.89). Relationship quality at T1 and T2 are composites of standardized scores on relationship satisfaction (M_T1_ = 4.55, SD_T1_ = 0.66, α_T1_ = 0.94; M_T2_ = 4.33, SD_T2_ = 0.83, α_T2_ = 0.97), commitment (M_T1_ = 7.12, SD_T1_ = 1.09, α_T1_ = 0.80; M_T2_ = 7.77, SD_T2_ = 1.78, α_T2_ = 0.88), and closeness (M_T1_ = 4.40, SD_T1_ = 0.58, r_T1_ = 0.41; M_T2_ = 4.32, SD_T2_ = 0.78, r_T2_ = 0.82) averaged at each time. Therefore, the Ms and SDs of CEM, relationship quality at T1 and T2 were omitted. Male is coded as 0 while female as 1. Please note that commitment at Time 2 was measured on a 9-point scale (1 = not at all, 9 = completely) rather than an 8-point scale (1 = not at all, 8 = completely).*

*^b^Sample size N = 58 dyads except for relationship quality at T2, N = 56 dyads.*

*p < 0.05, **p < 0.01, ***p < 0.001.

#### Childhood Emotional Maltreatment as a Predictor of Change in Compassionate Goals Over Time

CEM predicted decreased compassionate goals from T1 to T2 (β = −0.16, 95% CI [−0.31, −0.02], *p* = 0.029). The effect remained significant when we included actors’ attachment anxiety, attachment avoidance, hostility, depression, relationship quality, and social desirability at T1 together in the same analysis (β = −0.17, 95% CI [−0.32, −0.02], *p* = 0.028) as well as in separate analyses (see Supplementary Table 1.2). Thus, CEM explains variance in decreased compassionate goals that is unrelated to negative mechanisms, initial relationship quality, or social desirability.

#### Childhood Emotional Maltreatment as a Predictor of Change in Relationship Quality Through Change in Compassionate Goals

We used the data from the 56 pairs who were still with their partners at T2 to test the proposed path model. Results replicated the major finding in Study 1 that CEM predicted decreased compassionate goals over 2 months (β = −0.19, 95% CI [−0.34, −0.05], *p* = 0.011), which, in turn, predicted decreased relationship quality over 2 months (β = 0.26, 95% CI [0.13, 0.38], *p* < 0.001)^6^. Because CEM did not predict change in relationship quality (β = 0.04, 95% CI [−0.05, 0.14], *p* > 0.25), the results did not support an alternative model in which CEM predicted change in compassionate goals through change in relationship quality.

##### The Role of Relationship Length

We again explored whether relationship length moderated the key effects tested previously. Relationship length did not moderate the path between CEM and decreased compassionate goals (β = 0.07, 95% CI [−0.07, 0.21], *p* > 0.25).

##### Alternative Explanations

The path from decreased compassionate goals to decreased relationship quality remained significant when we included attachment anxiety, attachment avoidance, hostility, and depression as simultaneous mediators with decreased compassionate goals in a single analysis. Decreased compassionate goals still predicted decreased relationship quality (β = 0.27, 95% CI [0.15, 0.40], *p* < 0.001). In sum, a robustness check indicated that the path from CEM to decreased relationship quality through decreased compassionate goals was not due to attachment anxiety or avoidance, hostility, or depression.

#### Partners’ Compassionate Goals as a Protective Factor

**FIGURE 1:**
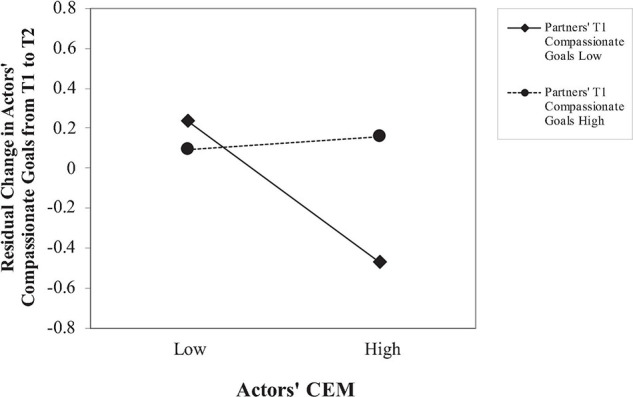
Actors’ CEM on change in actors’ compassionate goals from T1 to T2, plotted at 1 standard deviation above and below the mean on partners’ compassionate goals.

To test whether partners’ compassionate goals attenuate the association between actors’ CEM and actors’ decreased compassionate goals, we entered partners’ T1 compassionate goals, actors’ CEM, and their interaction as predictors of residual change in actors’ compassionate goals from T1 to T2. Actors’ CEM predicted change in actors’ compassionate goals (β = −0.16, 95% CI [−0.31, −0.02], *p* = 0.026); partners’ compassionate goals did not (β = 0.12, 95% CI [−0.02, 0.26], *p* = 0.10). As hypothesized, partners’ T1 compassionate goals moderated the association between actors’ CEM and change in actors’ compassionate goals (β = 0.19, 95% CI [0.04, 0.34], *p* = 0.014). Actors’ CEM predicted actors’ decreased compassionate goals when partners were lower in compassionate goals (β = −0.35, 95% CI [−0.56, −0.15], *p* = 0.001), but not when partners were higher in compassionate goals (β = 0.03, 95% CI [−0.18, 0.24], *p* > 0.25; see Figure 1).

### Discussion

Study 2 replicated the major findings of Study 1. CEM predicted declines in compassionate goals toward romantic partners over time, which predicted decreased relationship quality. These associations remained when controlling for relationship duration and negative characteristics of survivors previously linked to CEM (i.e., attachment anxiety and avoidance, hostility, and depression). The alternative possibility that CEM survivors’ relationship quality declines over time, which in turn predicts their decreased compassionate goals was not supported.

Of particular interest, Study 2 also found that when partners had low compassionate goals at T1, actors’ CEM predicted actors’ declining compassionate goals. However, when partners had high compassionate goals at T1, actors’ CEM did not predict change in actors’ compassionate goals. Thus, partners’ high compassionate goals appeared to buffer the association between actors’ CEM and declining compassionate goals over time.

## General Discussion

Existing research on the intimate relationships of CEM survivors focuses on how negative characteristics of survivors affect their relationships. The present research examined whether CEM predicts declines in a positive process—compassionate goals—in adult romantic relationships. In two studies, we found that CEM predicted decreasing compassionate goals, which in turn predicted declining relationship quality over time. Compassionate goals explain unique variance in decreased relationship quality above and beyond negative characteristics of survivors that have been shown to mediate this link, including attachment anxiety and avoidance, hostility, and depression. Neither study supported an alternative model in which CEM predicted decreased compassionate goals through decreased relationship quality.

These findings extend research on CEM. In our non-clinical samples, results indicate that people who experienced more childhood emotional maltreatment have difficulty sustaining compassionate goals toward their romantic partners. To our knowledge, this is one of the first investigations to examine whether self-reported CEM predicts decreased positive relationship processes. Our findings suggest that separately from effects of relationship insecurity, hostility, and depression, CEM predicts decreased intentions to support romantic partners, which undermines relationship quality.

The present research may help explain the higher rate of relationship dissolution among people who report CEM (Mullen et al., 1996). Past research showed that decreased positivity in relationships is a better predictor of relationship dissolution than negativity (Huston et al., 2001). Exploratory analyses in Study 1 found that decreased compassionate goals accounted for the association between CEM and relationship dissolution at T2. Thus, decreases in positive processes may contribute to relationship dissolution among CEM survivors. More research is needed to confirm this association.

Future research should further explore associations between CEM and positive relationship processes in adulthood. Declines in positive processes may be contributing to outcomes of people who experience CEM independently of negative processes, as in the current research. Future research should also examine how CEM influences other positive processes demonstrated to be important in building close relationships. For example, processes such as responding enthusiastically to partners’ good news (i.e., capitalization; Gable et al., 2004) enhance relationship satisfaction for both people in a dyadic relationship. Investigating whether CEM predicts declining compassionate goals specifically, or whether it also predicts declines in other positive mechanisms would afford a more complete picture of CEM survivors’ experiences in their relationships.

### Partners’ Positive Roles

Most research on how partners affect survivors of childhood maltreatment has focused on re-victimization by partners in adulthood (McMahon et al., 2015; see Li et al., 2019, for a meta-analysis). Less attention has been given to partners’ potentially positive roles. The present findings point to the positive role partners can play in relationships of CEM survivors, adding to the limited body of research on this topic.

Study 2 found a protective effect of partners’ compassionate goals. The decline in compassionate goals seen in CEM survivors was not observed when their partners had high compassionate goals. This is one of the first studies to examine how partner’s benevolent intentions buffer the effects of CEM. Thus, CEM survivors are not destined to have poor relationships. When partners have high compassionate goals, CEM survivors’ compassionate goals remain stable over time, potentially maintaining their level of relationship well-being. Future research on the positive role of partners in the lives of childhood maltreatment survivors is warranted.

### Implications for Research on Compassionate Goals

The present studies are also among the first to examine precursors of compassionate goals. In addition to replicating the positive association between compassionate goals and relationship quality from past research (see Crocker and Canevello, 2016, for a review), we found in both studies that CEM was associated with declining compassionate goals toward partners. This finding suggests that perceived adversity in early life interferes with people’s ability to sustain compassionate goals in their adult close relationships. Future research should examine why people with CEM decline in compassionate goals toward their romantic partners and whether CEM predicts declines in other prosocial processes.

This research also suggests that the beneficial effect of compassionate goals extended to this vulnerable population. Our finding regarding the stabilizing effect of partners’ compassionate goals for CEM survivors corroborates recent evidence of positive effects of compassionate goals on people with mood disorders (Erickson et al., 2018). Clinically depressed and/or anxious participants perceived higher support and had lower symptoms on days when they pursued compassionate goals. Moreover, when these participants perceived that their partners had high compassionate goals, their relationship satisfaction increased (Erickson et al., 2018). Taken together, these findings illustrate the power of compassionate goals, even in vulnerable populations.

### Limitations

The longitudinal design of the current research is correlational; manipulating CEM would be unethical. Thus, this research does not permit causal inferences. The longitudinal design of the present studies does enable tests of whether current reports of CEM predict change in compassionate goals and relationship quality over time, supporting the plausibility of a causal association (Kenny, 1975). Moreover, several alternative explanations failed to account for the association between CEM and change in compassionate goals over time. Furthermore, results point to the implausibility of an alternative process in which CEM predicts declines in relationship quality which, in turn, predicts decreased compassionate goals. Future research may also benefit from including an additional time point of 6 months or a year later to inform how these associations change over an even longer period of time.

Another limitation of these studies concerns the self-report nature of our measure of childhood maltreatment. Objective records of this type of childhood maltreatment are particularly difficult to obtain. According to a recent review (Dodge Reyome, 2010) and a meta-analysis (Norman et al., 2012), retrospective reports are used in about 98% of studies of CEM. Furthermore, childhood adversity should affect adults through current interpretations of early experience, independent of actual experience. Self-reported maltreatment relates to poor health and behavioral outcomes regardless of its concordance with presumably objective case records (e.g., Shaffer et al., 2008). In addition, a review of childhood abuse measures, including the CTQ, concluded that retrospective measures can detect a history of adversity (Hardt and Rutter, 2004; see also Bernstein et al., 1994; Bernstein and Fink, 1998). These findings suggest that self-reports of CEM have predictive validity.

Also, given the relatively small sample size, Study 2 was underpowered for tests of mediation and moderation. Future studies with sufficient power should replicate the mediation model tested in Study 2 and examine the indirect effect using tools such as MEDyad (Coutts et al., 2019). In addition, although the finding that partners’ compassionate goals moderate the link between actors’ CEM and actors’ compassionate goals supports theoretically derived hypotheses, future research should attempt to replicate this effect.

Finally, participants in the present studies were a non-clinical sample of mainly young, White, heterosexual college students, which may limit generalizability to other populations.

## Conclusion

The present results indicate the importance of studying how childhood adversities affect positive interpersonal processes such as compassionate goals. They also shed new light on the positive roles partners may play in the relationships of CEM survivors. Examining why and when people have difficulty sustaining positive intentions and behaviors affords a deeper understanding of close relationships among vulnerable populations, pointing to potential avenues for intervention.

## Data Availability Statement

The datasets presented in this article are not readily available because the consent form that participants signed stated that only members of the research team would have access to the dataset. Requests to access the datasets should be directed to the PI for the project, JC, crocker.37@osu.edu.

## Ethics Statement

The studies involving human participants were reviewed and approved by an Institutional Review Board at The Ohio State University. The participants provided their written informed consent to participate in this study.

## Author Contributions

LS and JC conceptualized the research idea and wrote the manuscript. JC and AC designed the research. KL performed the research. LS and JL analyzed the data. LS, JC, AC, and KL revised the manuscript. All authors contributed to the article and approved the submitted version.

## Conflict of Interest

The authors declare that the research was conducted in the absence of any commercial or financial relationships that could be construed as a potential conflict of interest.

## Publisher’s Note

All claims expressed in this article are solely those of the authors and do not necessarily represent those of their affiliated organizations, or those of the publisher, the editors and the reviewers. Any product that may be evaluated in this article, or claim that may be made by its manufacturer, is not guaranteed or endorsed by the publisher.

## Footnotes

1 Canevello, A., Jiang, T., and Crocker, J. (2021). *Relationships in the Ecosystem: Constructive Approaches to Interpersonal Problems and Their Consequences for Relationships.* Manuscript Submitted for Publication.

2 Jiang, T., Canevello, A., and Crocker, J. (2021). *How Relationships Foster Thriving: Compassionate Goals and Relationship Security Independently Predict Dispositional Growth Seeking Through Perceived Social Support*. Manuscript Submitted for Publication.

3 This sample consists of 121 complete cases and 22 cases with incomplete data. The Little’s MCAR test indicated that the pattern of missing values was completely random, χ^2^(2,367) = 2,431.40, *p* = 0.17 (Little, 1988). Out of 120 items, 28 have missing values, each of which only has 1 to 3 missing values (*M* = 1.25, *SD* = 0.52). Therefore, we replaced the missing items with the mean of the available non-missing items from those relevant subscales as recommended by Graham et al. (2003).

4 Note that commitment at Time 2 was measured on a 9-point scale (1 = *not at all*, 9 = *completely*) rather than an 8-point scale (1 = *not at all*, 8 = *completely*) as in Study 1.

5 This sample consisted of 99 complete cases and 17 cases with incomplete data. The Little’s MCAR test indicated that the pattern of missing values was completely random, χ^2^(1,884) = 1,472.35, *p* > 0.25 (Little, 1988). Out of 127 items, 20 have missing values, each of which only has 1 or 2 missing values (*M* = 1.15, *SD* = 0.36). Therefore, we replaced the missing items with the mean of the available non-missing items from those relevant subscales as recommended by Graham et al. (2003).

6 We did not test the indirect effect due to insufficient power for tests using tools like MEDyad Macro (Coutts et al., 2019).
