# Supplementary material for: Childhood Emotional Maltreatment and Romantic Relationships: The Role of Compassionate Goals
Source: Front Psychol. 2021 Nov 29;12:723126. doi: 10.3389/fpsyg.2021.723126 (PMC8666543; doi:10.3389/fpsyg.2021.723126)
Supplement: Supplementary file 1 [file Data_Sheet_1.docx]

Supplemental Online Materials

for

Childhood Emotional Maltreatment and Romantic Relationships: The Role of Compassionate Goals

## *1. CEM predicts decreased compassionate goals when controlling covariates*

The tables below show that the effect of CEM predicting decreased compassionate goals from T1 to T2 remain significant when we controlled for attachment anxiety, attachment avoidance, hostility, depression, relationship quality, and social desirability at T1 in separate analyses and together in the same analysis. Table S1.1 presents Study 1 results and Table S1.2 present Study 2 results.

Table S1.1

*Standardized Coefficient βs and 95% Confidence Intervals for T1 Variables Predicting Residual Change in Compassionate Goals Two Months Later in Study 1*

|  | Covariate entered individually | | | | Covariates entered together | |
| --- | --- | --- | --- | --- | --- | --- |
|  | Covariate with CEM | | CEM | | Covariate with CEM | |
|  | β | 95% CI | β | 95% CI | β | 95% CI |
| CEM |  |  |  |  | -.18* | [-.34, -.03] |
| Relationship quality | .17 | [-.01, .34] | -.16* | [-.31, -.02] | .15 | [-.08, .37] |
| Attachment anxiety | -.01 | [-.15, .14] | -.21** | [-.35, -.06] | .10 | [-.08, .27] |
| Attachment avoidance | -.13 | [-.27, .01] | -.19** | [-.33, -.05] | -.10 | [-.27, .07] |
| Hostility | -.01 | [-.15, .13] | -.21** | [-.35, -.07] | -.002 | [-.18, .17] |
| Depression | -.05 | [-.20, .10] | -.19** | [-.34, -.05] | -.03 | [-.21, .14] |
| Social desirability | -.02 | [-.16, .12] | -.22** | [-.36, -.07] | -.05 | [-.21, .11] |
| **p* < .05. ***p* < .01. | | | | | | |

Table S1.2

*Standardized Coefficient βs and 95% Confidence Intervals for Actors’ T1 Variables Predicting Residual Change in Actors’ Compassionate Goals Two Months Later in Study 2*

|  | Covariate entered individually | | | | |  | Covariates entered together | |
| --- | --- | --- | --- | --- | --- | --- | --- | --- |
|  | Covariate with Actors’ CEM | | | Actors’ CEM | |  | Covariate with Actors’ CEM | |
| Actors’ T1 Covariate | β | 95% CI |  | β | 95% CI |  | β | 95% CI |
| CEM |  |  |  |  |  |  | -.17* | [-.32, -.02] |
| Relationship quality | .19* | [.01, .38] |  | -.15* | [-.30, -.003] |  | .10 | [-.13, .32] |
| Attachment anxiety | -.16* | [-.31, -.01] |  | -.15* | [-.30, -.004] |  | -.15 | [-.31, .02] |
| Attachment avoidance | -.18* | [-.32, -.03] |  | -.15* | [-.29, -.01] |  | -.13 | [-.30, .04] |
| Hostility | .05 | [-.10, .21] |  | -.17* | [-.33, -.02] |  | .09 | [-.11, .28] |
| Depression | .02 | [-.14, .18] |  | -.17* | [-.32, -.02] |  | .02 | [-.16, .20] |
| Social desirability | -.06 | [-.21, .09] |  | -.17* | [-.32, -.02] |  | -.08 | [-.26, .09] |
| **p* < .05. | | | | | | | | |

## *2. Results for responsiveness as a mediator*

We conducted additional exploratory analyses in Study 1 to test whether CEM predicts change in compassionate goals through responsiveness. Results are presented below.

**Responsiveness*.*** Participants rated their responsiveness toward partners and their perception of partners’ responsiveness at T1. Responsiveness toward partners was measured with a 6-item scale (e.g., “I behaved warmly toward my partner”) and a parallel 6-item scale measured perception of partners’ responsiveness (e.g., “My partner behaved warmly toward me”) as in previous research (Crocker et al., 2017). Items began with the phrase, “In the past two weeks, in my relationship with my romantic partner” and were rated on 5-point scales (1 = *strongly disagree*; 5 = *strongly agree*). We standardized and averaged the two scale scores (*r =* .68, *p <* .001) to create a composite of responsiveness at T1.

## *Do Responsiveness and Change in Compassionate Goals Sequentially Mediate the Association between CEM and Change in Relationship Quality?*

We tested whether T1 responsiveness and residual change in compassionate goals from T1 to T2 sequentially mediated the association between T1 CEM and residual change in relationship quality in the 132 participants who were still with their partners at T2 using Model 6 in PROCESS (Hayes, 2013) with 10,000 bias-corrected bootstrapped samples. As hypothesized, CEM was associated with lower T1 responsiveness (β = -.27, *t*(130) = -3.55, 95% CI [-.42, -.12], *p* < .001), which predicted decreased compassionate goals (β = .16, *t*(129) = 2.03, 95% CI [.004, .31], *p* = .045), which in turn predicted decreased relationship quality over time (β = .36, *t*(128) = 4.52, 95% CI [.20, .52], *p* < .001; indirect effect = -.02, 95% CI [-.08, -.0003]; See Figure 1).

We also tested an alternative model in which CEM sequentially predicted T1 responsiveness and deteriorating relationship quality over time, which in turn predicted decreased compassionate goals toward partners. The alternative model was not supported (indirect effect = -.01, 95% CI [-.06, .002]).

## *Do Responsiveness and Change in Compassionate Goals Sequentially Mediate the Association between CEM and Relationship Dissolution at T2?*

To understand whether positive mechanisms can potentially predict CEM victims’ relationship dissolution, we conducted exploratory analyses. We tested whether T1 responsiveness and decreased compassionate goals sequentially mediate the association between CEM and relationship dissolution at T2. The analysis was based on all participants, including 11 participants who broke up with their partners between T1 and T2. Using Model 6 in PROCESS Version 2, which accommodates dichotomous outcomes (Hayes, 2013), with 10,000 bias-corrected bootstrapped samples, the mediation model explained 35.6% of the variance in relationship dissolution (Nagelkerke *R*^2^). CEM sequentially predicted T1 responsiveness (β = -.27, *t*(141) = -3.64, 95% CI [-.41, -.12], *p* < .001) and decreased compassionate goals (β = .20, *t*(140) = 2.55, 95% CI [.04, .35], *p* = .012), which in turn predicted the increased likelihood of relationship dissolution (β = -1.24, Z = -3.44, 95% CI [-1.95, -.54], *p* < .001; indirect effect = .07, 95% CI [.003, .23]).

The total effect of CEM on relationship status at T2 was also statistically significant with an odds ratio (Exp(B)) of 1.95, indicating that when CEM is raised by one unit, people are 1.95 times more likely to break up with their partners (*B* = .67, *SE* = .26, Wald(1) = 6.66, *p* = .01, 95% CI for Exp(B) [1.18, 3.25]).

## *3. Results for controlling other forms of maltreatment*

We conducted additional analyses in both studies to explore the relationships between different forms of maltreatment and test whether CEM predicts change in compassionate goals above and beyond other forms of childhood maltreatment. The results are presented below.

**Study 1**

Table S3.1

*Means, Standard Deviations, Minimum, Maximum, and Correlations among Study 1 Maltreatment Variables*

|  |  | *M* | *SD* | *Min* | *Max* | 1 | 2 | 3 | 4 |
| --- | --- | --- | --- | --- | --- | --- | --- | --- | --- |
| 1 | Emotional Abuse | 7.61 | 3.82 | 5 | 24 | — |  |  |  |
| 2 | Emotional Neglect | 7.92 | 3.26 | 5 | 19 | .62*** | — |  |  |
| 3 | Physical Abuse | 5.80 | 1.41 | 5 | 11 | .37*** | .15 | — |  |
| 4 | Physical Neglect | 6.29 | 2.19 | 5 | 18 | .35*** | .31*** | .12 | — |
| 5 | Sexual Abuse | 5.56 | 2.47 | 5 | 25 | .22** | .20* | .11 | -.02 |
| *Note*. **p* < .05. ***p* < .01. ****p* < .001. | | | | | | | | | |

## *CEM as a Predictor of Change in Compassionate Goals over Time*

When we regressed residual change in compassionate goals on CEM controlling for other three forms of childhood maltreatment at T1, CEM predicted decreased compassionate goals from T1 to T2, β = -.21, *t*(138) = -2.66, 95% CI [-.37, -.06], *p* = .009.

CEM still significantly predicted decreased compassionate goals even when we controlled childhood physical abuse, childhood physical neglect, childhood sexual abuse, relationship quality, attachment anxiety, attachment avoidance, hostility, depression, and social desirability at T1 together in a single analysis, β = -.18, *t*(132) = -2.02, 95% CI [-.36, -.003], *p* = .046.

**Study 2**

Table S3.2

*Means, Standard Deviations, Minimum, Maximum, and Correlations among Study 2 Maltreatment Variables*

|  |  | *M* | *SD* | *Min* | *Max* | 1 | 2 | 3 | 4 |
| --- | --- | --- | --- | --- | --- | --- | --- | --- | --- |
| 1 | Emotional Abuse | 7.24 | 3.47 | 5 | 24 | — |  |  |  |
| 2 | Emotional Neglect | 7.69 | 3.36 | 5 | 19 | .70*** | — |  |  |
| 3 | Physical Abuse | 6.28 | 2.92 | 5 | 23 | .73*** | .55*** | — |  |
| 4 | Physical Neglect | 6.04 | 1.73 | 5 | 14 | .50*** | .60*** | .47*** | — |
| 5 | Sexual Abuse | 5.43 | 2.04 | 5 | 21 | .24** | .03 | .26** | .09 |
| *Note*. ***p* < .01. ****p* < .001. | | | | | | | | | |

## *CEM as a Predictor of Change in Compassionate Goals over Time*

When we regressed residual change in compassionate goals on CEM controlling for other three forms of childhood maltreatment at T1, the relationship between CEM and change in compassionate goals from T1 to T2 is β = 18, 95% CI [-.02, 38], *p* = .071 while physical abuse and physical neglect significantly predicted decreased compassionate goals from T1 to T2 (physical abuse: β = -.29, 95% CI [-.48, -11], *p* = .002; physical neglect: β = -.26, 95% CI [-.43, -10], *p* = .002).

The flipped sign of the relationship between CEM and change in compassionate goals is likely due to the relatively high correlations between emotional maltreatment and physical maltreatment.

## *4. Results for controlling self-image goals*

We conducted additional analyses in both studies to explore whether the relationships between CEM and major variables still hold controlling self-image goals. The results are presented below.

**Study 1**

## *CEM as a Predictor of Change in Compassionate Goals over Time*

When we regressed residual change in compassionate goals on CEM controlling for self-image goals at T1, CEM predicted decreased compassionate goals from T1 to T2, β = -.20, *t*(141) = -2.88, 95% CI [-.34, -.06], *p* = .005, indicated that self-image goals did not account for the association between CEM and decreased compassionate goals.

CEM still significantly predicted decreased compassionate goals even when we controlled self-image goals, relationship quality, attachment anxiety, attachment avoidance, hostility, depression, and social desirability at T1 together in a single analysis, β = -.19, *t*(135) = -2.38, 95% CI [-.34, -.03], *p* = .019. This indicated that attachment anxiety and avoidance, hostility, depression, initial level of relationship quality, self-image goals, and social desirability did not account for the association between CEM and decreased compassionate goals.

## *CEM as a Predictor of Change in Relationship Quality through Change in Compassionate Goals*

We repeated the mediation analysis, adding attachment anxiety, attachment avoidance, hostility, depression, and self-image goals as simultaneous mediators with compassionate goals in a single analysis using Model 4 in PROCESS with 10,000 bias-corrected bootstrapped samples. The indirect effect through decreased compassionate goals remained (indirect effect = -.05, 95% CI [-.15, -.005]).

**Study 2**

## *CEM as a Predictor of Change in Compassionate Goals over Time*

When we regressed residual change in compassionate goals on CEM controlling for actors’ self-image goals at T1, CEM predicted decreased compassionate goals from T1 to T2, β = -.16, 95% CI [-.31, -.02], *p* = .027, indicated that self-image goals did not account for the association between CEM and decreased compassionate goals.

CEM still significantly predicted decreased compassionate goals even when we controlled actors’ self-image goals, relationship quality, attachment anxiety, attachment avoidance, hostility, depression, and social desirability at T1 together in a single analysis, β = -.17, 95% CI [-.32, -.03], *p* = .023. This indicated that actors’ attachment anxiety and avoidance, hostility, depression, initial level of relationship quality, self-image goals, and social desirability did not account for the association between CEM and decreased compassionate goals.

## *CEM as a Predictor of Change in Relationship Quality through Change in Compassionate Goals*

The path from decreased compassionate goals to decreased relationship quality remained significant when we included self-image goals, attachment anxiety, attachment avoidance, hostility, and depression as simultaneous mediators with decreased compassionate goals in a single analysis. Decreased compassionate goals still predicted decreased relationship quality (β = .29, 95% CI [.16, .42], *p* < .001).

## *5. Means, SDs, and t-tests comparing males and females*

We conducted additional t-tests in both studies to examine gender differences on the major variables. None of the gender differences was significant. The results are presented below.

**Study 1**

Table S5.1

*Means, SD, and t-tests comparing Males and Females on Study 1 Variables*

|  | Male | |  | Female | | *t*-test |
| --- | --- | --- | --- | --- | --- | --- |
|  | *M* | *SD* |  | *M* | *SD* |  |
| CEM^a^ | -.09 | .85 |  | .03 | 1.04 | -.58 |
| Compassionate goals (T1) | 4.26 | .52 |  | 4.15 | .53 | 1.03 |
| Compassionate goals (T2) | 4.10 | .66 |  | 4.01 | .72 | .61 |
| Relationship quality (T1^a^) | .06 | .73 |  | -.02 | .85 | .45 |
| Relationship quality (T2^a, b^) | .12 | .71 |  | -.03 | .96 | .80 |
| Attachment Anxiety (T1) | 1.98 | .70 |  | 2.21 | .91 | -1.35 |
| Attachment Avoidance (T1) | 1.53 | .55 |  | 1.57 | .56 | -.39 |
| Hostility (online pretest) | 2.02 | .70 |  | 2.31 | .81 | -1.89 |
| Depression (online pretest) | 1.72 | .53 |  | 1.89 | .53 | -1.65 |
| Social desirability | 15.75 | 5.79 |  | 16.31 | 4.74 | -.57 |
| Relationship length | 1.49 | 1.44 |  | 1.48 | 1.32 | .05 |
| Note: ^a^ CEM = Childhood Emotional Maltreatment. CEM is the standardized composite of emotional abuse and emotional neglect scores. Relationship quality at T1 and T2 are composites of standardized scores on relationship satisfaction, commitment, and closeness averaged at each time.  ^b^*N* = 143 (*N*_male_ = 34, *N*_female_ = 109) except for relationship quality at Time 2, *N* = 132 (*N*_male_ = 30, *N*_female_ = 102). | | | | | | |

**Study 2**

Table S5.2

*t-test Results Comparing Males and Females on Study 2 Variables*

|  | Male | |  | Female | | *t*-test |
| --- | --- | --- | --- | --- | --- | --- |
|  | *M* | *SD* |  | *M* | *SD* |  |
| CEM^a^ | -.15 | .72 |  | .15 | 1.20 | -1.67 |
| Compassionate goals (T1) | 4.19 | .52 |  | 4.28 | .44 | -1.04 |
| Compassionate goals (T2) | 4.08 | .64 |  | 4.23 | .53 | -1.38 |
| Relationship quality (T1^a^) | -.08 | .86 |  | .08 | .75 | -1.06 |
| Relationship quality (T2^a, b^) | -.12 | 1.00 |  | .12 | .70 | -1.45 |
| Attachment Anxiety (T1) | 2.01 | .85 |  | 2.17 | .97 | -.95 |
| Attachment Avoidance (T1) | 1.67 | .59 |  | 1.59 | .57 | .75 |
| Hostility (online pretest) | 2.20 | .80 |  | 2.09 | .74 | .75 |
| Depression (online pretest) | 1.76 | .63 |  | 1.81 | .49 | -.53 |
| Social desirability | 16.93 | 5.10 |  | 16.34 | 4.61 | .65 |
| Relationship length | 1.34 | 1.30 |  | 1.30 | 1.28 | .15 |
| Note: ^a^ CEM = Childhood Emotional Maltreatment. CEM is the standardized composite of emotional abuse and emotional neglect scores. Relationship quality at T1 and T2 are composites of standardized scores on relationship satisfaction, commitment, and closeness averaged at each time.  ^b^ Sample size *N* = 58 dyads except for relationship quality at T2, *N* = 56 dyads. | | | | | | |

## *6. Results for multiple imputation*

We conducted additional analyses in both studies to test whether the results change if we use multiple imputation rather than mean substitution as the method of handling missing data. Some analyses could not be conducted using multiple imputation. The results using multiple imputation are presented below.

## Study 1

## *Primary Measures*

**Childhood Emotional Maltreatment.** The Childhood Trauma Questionnaire (CTQ; Bernstein & Fink, 1998) assesses self-reports of maltreatment experienced during childhood. Participants rated ten statements from 1 (*never true*) to 5 (*very often true*) regarding their families prior to age 13, half on emotional abuse (e.g., “People in my family said hurtful or insulting things to me”; *M* = 7.59) and the rest on emotional neglect (e.g., “People in my family looked out for each other” (reverse scored); *M* = 7.92). We averaged emotional abuse and neglect scores (*r =* .62, *p <* .001; Lassri et al., 2016).

## Results

## *Overview*

Table S6.1 presents the means, standard deviations, coefficient alphas, and correlations among variables. As predicted, CEM correlated negatively with compassionate goals and relationship quality at both time points. Consistent with past research (Canevello & Crocker, 2010, Study 2), compassionate goals correlated positively with relationship quality at T1 and T2. Because gender was not related to any other variables, it was not included in the subsequent analyses.

After conducting descriptive analyses, we standardized all variables to obtain interpretable and comparable effect sizes (Schielzeth, 2010). We created residual change scores for compassionate goals and relationship quality by regressing the T2 variable on the T1 variable and saving the residuals as the indicator of residual change.

## *CEM as a Predictor of Change in Compassionate Goals over Time*

When we regressed residual change in compassionate goals on CEM, CEM predicted decreased compassionate goals from T1 to T2, β = -.20, *t*(141) = -2.89, 95% CI [-.34, -.07], *p* = .004. Then we examined whether this association was due to other variables by controlling for relationship quality, attachment anxiety, attachment avoidance, hostility, depression, and social desirability at T1, first in separate analyses and then together in a single analysis. The results showed that CEM still significantly predicted decreased compassionate goals (see Table S6.2). It indicated that attachment anxiety and avoidance, hostility, depression, initial level of relationship quality, and social desirability did not account for the association between CEM and decreased compassionate goals.

## Study 2

## Results

## *Overview*

Table S6.3 presents the means, standard deviations, coefficient alphas, and correlations for primary variables in Study 2. Analyses addressed two main questions: (a) Does CEM predict decreased compassionate goals over two months, which predicts decreased relationship quality as in Study 1? and (b) Do partners’ compassionate goals attenuate decreased compassionate goals in CEM victims?

In these data, individuals were nested within couples. We accounted for the nonindependence of individuals within dyads using the mixed command in SPSS and treating dyad members as distinguishable by specifying heterogenous compound symmetry covariance structure (Campbell & Kashy, 2002; Kenny et al., 2006). All variables were standardized to provide interpretable effect sizes. As in Study 1, we created residualized variables for change in compassionate goals and change in relationship quality.

Because gender was not correlated with any other variables, we did not include it in subsequent analyses. Partners’ T1 compassionate goals were uncorrelated with actor variables except attachment anxiety (*r* = -.19, *p* = .044).

## *CEM as a Predictor of Change in Compassionate Goals over Time*

CEM predicted decreased compassionate goals from T1 to T2 (β = -.16, 95% CI [-.30, -.03], *p* = .03). The effect remained significant when we controlled for actors’ attachment anxiety, attachment avoidance, hostility, depression, relationship quality, and social desirability at T1 in separate analyses and together in the same analysis (see Table S6.4). Thus, CEM explains variance in decreased compassionate goals that is unrelated to negative mechanisms, initial relationship quality, or social desirability.

## *CEM as a Predictor of Change in Relationship Quality through Change in Compassionate Goals*

We used the data from the 56 pairs who were still with their partners at T2 to test the proposed path model. Results replicated the major finding in Study 1 that CEM predicted decreased compassionate goals over two months (β = -.20, 95% CI [-.34, -.05], *p* = .01), which, in turn, predicted decreased relationship quality over two months (β = .23, 95% CI [.10, .35], *p* < .001). Because CEM did not predict change in relationship quality (β = .05, 95% CI [-.04, .14], *p* > .25), the results did not support an alternative model in which CEM predicted change in compassionate goals through change in relationship quality.

**The Role of Relationship Length*.*** We again explored whether relationship length moderated the key effects tested previously. The result showed that relationship length did not moderate the path between CEM and decreased compassionate goals (β = .07, 95% CI [-.07, .21], *p* > .25).

**Alternative Explanations*.*** The path from decreased compassionate goals to decreased relationship quality remained significant when we included attachment anxiety, attachment avoidance, hostility, and depression as simultaneous mediators with decreased compassionate goals in a single analysis. Decreased compassionate goals still predicted decreased relationship quality (β = .24, 95% CI [.12, .37], *p* < .001).

## *Partners’ Compassionate Goals as a Protective Factor*

To test whether partners’ compassionate goals attenuate the association between actors’ CEM and actors’ decreased compassionate goals, we entered partners’ T1 compassionate goals, actors’ CEM, and their interaction as predictors of residual change in actors’ compassionate goals from T1 to T2. The main effect of actors’ CEM was significant (β = -.16, 95% CI [-.24, -.09], *p* = .024); that of partners’ compassionate goals was not (β = .12, 95% CI [-.03, .26], *p* = .11). The interaction between actors’ CEM and partners’ T1 compassionate goals on change in actors’ compassionate goals was significant (β = .19, 95% CI [.11, .27], *p* = .013). Actors’ CEM predicted actors’ decreased compassionate goals over two months when partners were lower in compassionate goals (β = -.35, 95% CI [-.63, -.17], *p* = .001), but not when partners were higher in compassionate goals (β = .03, 95% CI [-.19, .27], *p* > .25; see Figure 1).

Table S6.1

*Means, Standard Deviations, Coefficient Alphas, and Correlations among Study 1 Variables*

|  |  | *M* | 1 | 2 | 3 | 4 | 5 | 6 | 7 | 8 | 9 | 10 | 11 |
| --- | --- | --- | --- | --- | --- | --- | --- | --- | --- | --- | --- | --- | --- |
| 1 | CEM^a^ | - |  |  |  |  |  |  |  |  |  |  |  |
| 2 | Compassionate goals (T1) | 4.18 | -.21* |  |  |  |  |  |  |  |  |  |  |
| 3 | Compassionate goals (T2) | 4.03 | -.31*** | .53*** |  |  |  |  |  |  |  |  |  |
| 4 | Relationship quality (T1^a^) | - | -.30*** | .37*** | .40*** |  |  |  |  |  |  |  |  |
| 5 | Relationship quality (T2^a, b^) | - | -.16 | .35*** | .53*** | .56*** |  |  |  |  |  |  |  |
| 6 | Attachment Anxiety (T1) | 2.16 | .30*** | -.17* | -.15 | -.48*** | -.19* |  |  |  |  |  |  |
| 7 | Attachment Avoidance (T1) | 1.56 | .16 | -.40*** | -.37*** | -.58*** | -.31*** | .42*** |  |  |  |  |  |
| 8 | Hostility (online pretest) | 2.24 | .17* | -.30*** | -.21* | -.15 | -.16 | .32*** | .26** |  |  |  |  |
| 9 | Depression (online pretest) | 1.85 | .34*** | -.23** | -.23** | -.33*** | -.11 | .46*** | .31*** | .47*** |  |  |  |
| 10 | Social desirability | 16.15 | -.28*** | .35*** | .22** | .19* | .14 | -.18* | -.18* | -.46*** | -.21* |  |  |
| 11 | Gender | - | .05 | -.09 | -.04 | -.05 | -.07 | .11 | .03 | .16 | .15 | .06 |  |
| 12 | Relationship length | 1.48 | -.08 | -.08 | -.10 | .20* | .07 | -.18* | -.10 | -.08 | .13 | .02 | -.004 |
| *Note*. ^a^ CEM = Childhood Emotional Maltreatment. CEM is the standardized composite of emotional abuse (*M* = 7.59) and emotional neglect scores (*M* = 7.92). Relationship quality at T1 and T2 are composites of standardized scores on relationship satisfaction (*M*_T1_ = 4.59; *M*_T2_ = 4.45), commitment (*M*_T1_ = 7.18; *M*_T2_ = 7.47,), and closeness (*M*_T1_ = 4.35; *M*_T2_ = 4.20) averaged at each time. Therefore, the *M*s and *SD*s of CEM, relationship quality at T1 and T2 were omitted.  Male is coded as 0 while female as 1.  ^b^*N* = 143 except for relationship quality at Time 2, *N* = 132.  **p* < .05. ***p* < .01. ****p* < .001. | | | | | | | | | | | | | |

Table S6.2

*Standardized Coefficient βs and 95% Confidence Intervals for T1 Variables Predicting Residual Change in Compassionate Goals Two Months Later in Study 1*

|  | Covariate entered individually | | | | Covariates entered together | |
| --- | --- | --- | --- | --- | --- | --- |
|  | Covariate with CEM | | CEM | | Covariate with CEM | |
|  | β | 95% CI | β | 95% CI | β | 95% CI |
| CEM |  |  |  |  | -.18* | [-.33, -.03] |
| Relationship quality | .20* | [.02, .37] | -.15* | [-.29, -.01] | .20 | [-.03, .42] |
| Attachment anxiety | -.003 | [-.15, .14] | -.20** | [-.34, -.06] | .11 | [-.06, .28] |
| Attachment avoidance | -.13 | [-.27, .01] | -.18** | [-.32, -.04] | -.08 | [-.25, .09] |
| Hostility | -.01 | [-.15, .13] | -.20** | [-.34, -.06] | -.02 | [-.19, .15] |
| Depression | -.04 | [-.19, .10] | -.19* | [-.33, -.04] | -.02 | [-.19, .15] |
| Social desirability | -.03 | [-.17, .12] | -.21** | [-.35, -.07] | -.06 | [-.22, .10] |
| **p* < .05. ***p* < .01. | | | | | | |

Table S6.3

*Means, Standard Deviations, Coefficient Alphas, and Correlations among Study 2 Variables*

|  |  | *M* | 1 | 2 | 3 | 4 | 5 | 6 | 7 | 8 | 9 | 10 | 11 |
| --- | --- | --- | --- | --- | --- | --- | --- | --- | --- | --- | --- | --- | --- |
| 1 | CEM^a^ | - |  |  |  |  |  |  |  |  |  |  |  |
| 2 | Compassionate goals (T1) | 4.24 | -.03 |  |  |  |  |  |  |  |  |  |  |
| 3 | Compassionate goals (T2) | 4.16 | -.17 | .55*** |  |  |  |  |  |  |  |  |  |
| 4 | Relationship quality (T1^a^) | - | -.02 | .42*** | .40*** |  |  |  |  |  |  |  |  |
| 5 | Relationship quality (T2^a, b^) | - | -.01 | .39*** | .53*** | .65*** |  |  |  |  |  |  |  |
| 6 | Attachment Anxiety (T1) | 2.09 | .08 | -.16 | -.25** | -.37*** | -.27** |  |  |  |  |  |  |
| 7 | Attachment Avoidance (T1) | 1.63 | .05 | -.50*** | -.45*** | -.53*** | -.52*** | .32*** |  |  |  |  |  |
| 8 | Hostility (online pretest) | 2.14 | .21* | -.24** | -.11 | -.24** | -.17 | .28** | .22* |  |  |  |  |
| 9 | Depression (online pretest) | 1.79 | .22* | -.18 | -.13 | -.22* | -.27** | .34*** | .15 | .54*** |  |  |  |
| 10 | Social desirability | 16.64 | -.07 | .27** | .10 | .24* | .18 | -.21* | -.19* | -.50*** | -.30*** |  |  |
| 11 | Gender | - | .16 | .08 | .12 | .14 | .14 | .09 | -.08 | -.07 | .06 | -.05 |  |
| 12 | Relationship length | 1.32 | .11 | .11 | .13 | .24** | .24* | -.02 | -.18* | .15 | .17 | -.10 | -.02 |
| *Note*. ^a^ CEM = Childhood Emotional Maltreatment. CEM is the standardized composite of emotional abuse (*M* = 7.22) and emotional neglect scores (*M* = 7.67). Relationship quality at T1 and T2 are composites of standardized scores on relationship satisfaction (*M*_T1_ = 4.55, *M*_T2_ = 4.33), commitment (*M*_T1_ = 7.00; *M*_T2_ = 7.23), and closeness (*M*_T1_ = 4.40; *M*_T2_ = 4.32) averaged at each time. Therefore, the *M*s and *SD*s of CEM, relationship quality at T1 and T2 were omitted. Male is coded as 0 while female as 1.  ^b^ Sample size *N* = 58 dyads except for relationship quality at T2, *N* = 56 dyads.  **p* < .05. ***p* < .01. ****p* < .001. | | | | | | | | | | | | | |

Table S6.4

*Standardized Coefficient βs and 95% Confidence Intervals for Actors’ T1 Variables Predicting Residual Change in Actors’ Compassionate Goals Two Months Later in Study 2*

|  | Covariate entered individually | | | | |  | Covariates entered together | |
| --- | --- | --- | --- | --- | --- | --- | --- | --- |
|  | Covariate with Actors’ CEM | | | Actors’ CEM | |  | Covariate with Actors’ CEM | |
| Actors’ T1 Covariate | β | 95% CI |  | β | 95% CI |  | β | 95% CI |
| CEM |  |  |  |  |  |  | -.17* | [-.32, -.02] |
| Relationship quality | .20* | [.01, .38] |  | -.16* | [-.30, -.01] |  | .09 | [-.13, .32] |
| Attachment anxiety | -.16* | [-.31, -.01] |  | -.15* | [-.30, -.01] |  | -.14 | [-.30, .03] |
| Attachment avoidance | -.18* | [-.33, -.04] |  | -.15* | [-.29, -.01] |  | -.13 | [-.31, .04] |
| Hostility | .05 | [-.11, .20] |  | -.17* | [-.25, -.10] |  | .08 | [-.12, .28] |
| Depression | .02 | [-.14, .17] |  | -.17* | [-.30, -.04] |  | .02 | [-.16, .21] |
| Social desirability | -.06 | [-.21, .09] |  | -.17* | [-.24, -.09] |  | -.08 | [-.26, .09] |
| **p* < .05. | | | | | | | | |

## *7. Other papers based on the same datasets*

Here we explain the differences between the current paper and those that have used the same datasets. We explain the timing of measures, the variables analyzed in different manuscripts, and the distinct research aims of the different manuscripts. Specifically, the purposes of other manuscripts based on these data differed from those of the present study. Importantly, no other studies from our lab, including other manuscripts based on these data sets, have analyzed CEM.

Three other manuscripts report findings from one or both of these two data sets: (1) Crocker, Canevello & Lewis, *JPSP* 2017, Studies 1 and 2; (2) Canevello & Crocker, *Motivation and Emotion*, 2017, Study 1; and (3) Canevello, Jiang & Crocker, in preparation, 2021, Study 5.

To understand how the measures from the present studies do and do not overlap with measures reported in other manuscripts, it is helpful to understand the structure of the two data sets. In both studies, participants first completed a battery of personality and individual difference measures online (T1a). Soon after, they came to the laboratory where they gave finger stick blood samples and a saliva sample and were attached to a heart rate monitor, then completed another battery of measures which mainly asked about their romantic relationship and health outcomes (T1b). They then completed a potentially threatening relationship task. In Study 1, this involved providing feedback to a romantic partner (who was not a participant in the study), which was recorded but not actually given to partners. In Study 2, this involved both partners having a discussion about a conflict in their relationship. Additional measures were collected after this relationship task (T1c). Participants were contacted approximately two months later and completed an additional series of measures (T2).

Of the variables included in the present studies, childhood emotional maltreatment (CEM) was assessed at T1a and is not included in any previous manuscript. Change in compassionate goals from T1b to T2 was included in Crocker et al. (2017, Study 1) to rule out the possibility that nonzero-sum beliefs predict change in goals (they did not). Change in relationship quality from T1b to T2 is included as an outcome in both of the present studies and in Study 1 of Crocker et al (2017, Study 1), although the research question—whether T1b compassionate goals predict change in nonzero-sum beliefs, which in turn predict change in relationship quality—differed. Crocker et al. (2017, Study 2) examined change in relationship quality from T1b to T1c (after a conflict discussion), which is not analyzed in the present studies.

Canevello & Crocker (2017, Study 1) analyzed the association between T1c compassionate goals (while providing feedback for partner) and T1c ecosystem emotions (clear, connected, peaceful, and loving) while providing feedback; neither of these variables is included in the present studies.

Canevello et al. (2021, in preparation) reports the development of a new measure of ecosystem approaches to conflict. Study 5 of that manuscript reports analyses of the data set used in Study 2 in the present manuscript. Specifically, it reports whether compassionate goals at T1b predict ecosystem approaches at T1b, which in turn predict T1c variables assessing the outcome of the discussion, including change in relationship quality from T1b to T1c. Although T1 compassionate goals and relationship quality are used in that study, Study 5 of Canevello et al. (2021) does not examine any T2 outcomes, which are the focus of the present manuscript.
